# Bridging gaps in the molecular phylogeny of the Lymnaeidae (Gastropoda: Pulmonata), vectors of Fascioliasis

**DOI:** 10.1186/1471-2148-10-381

**Published:** 2010-12-09

**Authors:** Ana C Correa, Juan S Escobar, Patrick Durand, François Renaud, Patrice David, Philippe Jarne, Jean-Pierre Pointier, Sylvie Hurtrez-Boussès

**Affiliations:** 1 Laboratoire Génétique et Evolution des Maladies Infectieuses, UMR 2724 CNRS-IRD, IRD 911 avenue Agropolis, BP64501, 34394 Montpellier Cedex 5, France; 2 Institut des Sciences de l'Evolution UMR 5554, Université Montpellier II, Place Eugène Bataillon, 34095 Montpellier Cedex 5, France; 3 Centre d'Ecologie Fonctionnelle et Evolutive UMR 5175, 1919 Route de Mende, Campus CNRS, 34293 Montpellier Cedex 5, France; 4 USR 3278 CNRS-EPHE, CRIOBE Université de Perpignan, 68860 Perpignan-Cedex, France; 5 Département de Biologie-Ecologie (Faculté des Sciences) cc- 046- Université Montpellier 2, 4 Place Eugène Bataillon, 34095 Montpellier Cedex 5, France

## Abstract

**Background:**

Lymnaeidae snails play a prominent role in the transmission of helminths, mainly trematodes of medical and veterinary importance (*e.g*., *Fasciola *liver flukes). As this family exhibits a great diversity in shell morphology but extremely homogeneous anatomical traits, the systematics of Lymnaeidae has long been controversial. Using the most complete dataset to date, we examined phylogenetic relationships among 50 taxa of this family using a supermatrix approach (concatenation of the 16 S, ITS-1 and ITS-2 genes, representing 5054 base pairs) involving both Maximum Likelihood and Bayesian Inference.

**Results:**

Our phylogenetic analysis demonstrates the existence of three deep clades of Lymnaeidae representing the main geographic origin of species (America, Eurasia and the Indo-Pacific region). This phylogeny allowed us to discuss on potential biological invasions and map important characters, such as, the susceptibility to infection by *Fasciola hepatica *and *F. gigantica*, and the haploid number of chromosomes (n). We found that intermediate hosts of *F. gigantica *cluster within one deep clade, while intermediate hosts of *F. hepatica *are widely spread across the phylogeny. In addition, chromosome number seems to have evolved from n = 18 to n = 17 and n = 16.

**Conclusion:**

Our study contributes to deepen our understanding of Lymnaeidae phylogeny by both sampling at worldwide scale and combining information from various genes (supermatrix approach). This phylogeny provides insights into the evolutionary relationships among genera and species and demonstrates that the nomenclature of most genera in the Lymnaeidae does not reflect evolutionary relationships. This study highlights the importance of performing basic studies in systematics to guide epidemiological control programs.

## Background

Basommatophora (Gastropoda: Pulmonata) is a suborder comprising essentially all pulmonate gastropods living in freshwater. Basommatophorans are monophyletic and encompass five families: Acroloxidae, Chilinidae, Lymnaeidae, Physidae, and Planorbidae (including the Ancylidae) [1]. The group contains ~300 species and has been extensively studied because some species have a role in transmitting parasites of human and veterinary importance (*e.g*., *Schistosoma *and *Fasciola*). The Lymnaeidae, Physidae and Planorbidae comprise ~90% of the Basommatophoran species [1,2]. The phylogenetic relationships within the Physidae and Planorbidae are now well established (*e.g*., [3-5]). However, the phylogeny of the Lymnaeidae has only been partially inferred [6-11] and we currently lack a comprehensive treatment of this family.

Lymnaeidae snails are distributed worldwide [12-14]. They are of major medical and veterinary importance since they act as vectors of parasites that severely affect human populations and livestock, and cause important economic losses [15,16]. Indeed, lymnaeids serve as intermediate hosts of at least 71 trematode species distributed among 13 families [17,18], including some species of Schistosomatidae and Echinostomatidae, with implications for human health [19,20], and *Paramphistomum daubneyi*, which is of veterinary interest [21]. Undoubtedly, the most emblematic case of parasite transmitted by lymnaeids is *Fasciola hepatica *(Digenea: Fasciolidae), the agent of fascioliasis. The disease it causes is recognized as a major veterinary problem as it is responsible of the loss of productive capacity (*e.g*., meat, milk). Fascioliasis is also an important human disease with about 20 million cases around the world [22]. *Fasciola hepatica *presumably originates from Europe and now has a worldwide distribution. The definitive hosts of this parasite are vertebrates, usually mammals, *e.g*., cows, sheep, goats, buffalos, and also humans. Mollusks, generally lymnaeids, are required as intermediate hosts to complete the life cycle. At least 20 species of Lymnaeidae have been described as potential vectors of fascioliasis (see reviews in [17,23,24]). Due to the important role of these species as intermediate hosts of trematodes, a solid phylogenetic framework of Lymnaeidae is required. The correct identification of intermediate-host species should help characterize areas of epidemiological risk and increase our understanding of the evolution of the Lymnaeidae- *Fasciola *host-parasite interaction.

Lymnaeidae exhibit a great diversity in shell morphology which is linked to substantial eco-phenotypic plasticity (see *e.g*., [25,26]). Hubendick [12] illustrated this point by compiling up to 1143 species names, a large number of which he synonymized. In contrast, the anatomy of their reproductive tracts (including prostate, penis and preputium) is extremely homogeneous (*e.g*., [1,27,28]). Immunological [29], cytogenetical [30,31], enzyme electrophoresis studies [25,32,33], and DNA-based approaches [7,10] have demonstrated extensive homoplasy in anatomical characters [12,26,34-38]. The difference between patterns in shell morphology and anatomy explains why lymnaeid systematics has been controversial [6]. Today, it is accepted that the total number of species might be less than 100 [1], with most occurring in the Palearctic and Nearctic regions [2]. Although some effort has been done to resolve their phylogenetic relationships, the small number of genes or species considered (*e.g*., a single gene in [7]) and limited geographical coverage (*e.g*., Neotropic in [10], Australasia in [11] and ancient European lakes in [39]) represent severe limitations and biases. Furthermore, some well-recognized species have never been considered (*e.g*., the Neotropical species *Lymnaea cousini *and *L. diaphana*). These limitations and biases have generated gaps in our understanding of their biogeographic patterns, and their epidemiology in areas of high endemicity of trematode diseases (*e.g*., South America; [40]).

In this paper, we contribute to fill these gaps by performing a phylogenetic analysis using a supermatrix approach. We infer the most complete phylogeny to date in Lymnaeidae using sequences of 50 taxa (*i.e*., approximately half of the supposed diversity of the family) covering most Neotropical species (including the unstudied *L. diaphana*, *L. cousini *and *Lymnaea *sp. from Colombia). Using this phylogenetic framework, we analyze how susceptibility to infection by *F. hepatica *and *F. gigantica *(the sister-species of *F. hepatica *mainly responsible for fascioliasis in Asia and Africa) has evolved. In addition, this phylogeny allows us to establish biogeographic aspects, to pinpoint potential biological invasions, and determine the evolution of chromosome numbers.

## Methods

### Sampling

Sequences of one to three genes among the two nuclear internal transcribed spacers of the ribosomal DNA (ITS-1 and ITS-2) and the mitochondrial 16 S ribosomal DNA of 38 lymnaeid species and two outgroups, the planorbids *Bulinus forskalii *and *Biomphalaria tenagophila *(Planorbidae), were retrieved from GenBank (Table 1). We chose these three genes because they are highly variable and have been extensively used in phylogenetic studies of Lymnaeidae. They represent most known clades of this family (*e.g*., [6,7,9-11,41]). In addition, living individuals of 12 lymnaeid species and an additional outgroup, *Physa acuta *(Physidae), were collected in 13 populations sampled in a variety of countries/continents (Table 1). Sampled individuals were stored in 80% ethanol for DNA analyses.

**Table 1:** Names and accession numbers given in GenBank for the species used in the phylogeny presented in figure 1.

Species	Country, locality	ITS-1	ITS-2	16S
*Autropeplea lessoni*	Australia	NA	EU556308	EU556266
*Autropeplea ollula*	Philippines	NA	NA	U82067
*Autropeplea tomentosa*	Australia, Guyra	NA	EU556270	AF485645
*Autropeplea viridis *(= *Lymnaea viridis*)	Australia, Perth (Queensland)	NA	EU556313	AF485642
*Biomphalaria tenagophila*	Brazil, Rio Grande do Sul, Goias	AY425730	AF198655	AY030220
*Bulimnea megasoma*	Canada, Manitoba	NA	NA	U82069
*Bulinus forskalii*	Tanzania, Mafia Island, Angola, Quifangondo	AF503573	AM921961	AY029550
*Bullastra cumingiana*	Philippines, Luzon	NA	EU556314	U82068
*Fossaria bulimoides*	USA, Oklahoma	NA	NA	AF485657
*Fossaria obrussa*	Canada, Ontario	NA	NA	AF485658
***Galba truncatula***	France, Limoges	HQ283251	HQ283262	HQ283236
*Kutikina hispida*	Australia, Franklin River	NA	EU556311	EU556268
*Lymnaea corvus*	Austria, Wallersee;	NA	AJ319625	U82079
(= *Stagnicola corvus*)	Bulgaria			
***Lymnaea cousini***	Venezuela, Mucubají	HQ283255	HQ283266	HQ283237
***Lymnaea cubensis***	Colombia, Antioquia	HQ283253	HQ283264	FN182204
(= *Bakerilymnaea cubensis*)				
***Lymnaea diaphana***	Argentina, Lago Escondido	HQ283256	HQ283260	HQ283241
*Lymnaea fuscus*	Germany, Westfallen	AJ626855	AJ319622	NA
*Lymnaea *gen. sp.	Hawaii, Kauai	NA	NA	U82070
*Lymnaea humilis*	USA, Charleston (South Carolina)	FN182193	FN182191	FN182195
***Lymnaea *(*Radix*) *natalensis***	La Réunion, Bras de Pontho	HQ283257	HQ283270	HQ283242
*Lymnaea neotropica*	Peru, Lima	AM412228	AM412225	NA
*Lymnaea occulta*	Poland	AJ626858	AJ457042	NA
(= *Catascopia occulta*)				
***Lymnaea palustris***	Sweden, Umea;	HQ283250	HQ283267	U82082
(= *Stagnicola palustris*)	Germany			
***Radix peregra***	France, Viols le Fort (Herault); Turkey, Söke	HQ283258	HQ283271	U82074
*Lymnaea *sp. EEAR-China-2002	China, Wuhan	NA	NA	AF485643
***Lymnaea *sp. Colombia**	Colombia, Antioquia	HQ283252	HQ283263	HQ283235
*Lymnaea *sp. EEAR-Hawaii-2002	USA, Hawaii	NA	NA	AF485644
***Lymnaea stagnalis***	France, Lacépede (Lot et Garonne); Canada	NA	HQ283268	AF485659
*Stagnicola turricola*	Austria, Wallersee	AJ626853	AJ319618	NA
(= *Lymnaea palustris turricola*)				
***Lymnaea viatrix***	Argentina, Rio Negro	HQ283254	HQ283265	HQ283239
***Omphiscola glabra***	France, Limoges	HQ283249	HQ283269	HQ283246
***Physa acuta***	Mexico, Veracruz	HQ283259	HQ283272	GQ415021
***Pseudosuccinea columella***	Colombia, Antioquia; Australia	HQ283248	HQ283261	U82073
*Radix ampla*	Austria, Wallersee	NA	AJ319640	NA
*Radix auricularia*	Czech Republic; Danube Delta, Romania	NA	AJ319628	AF485646
*Radix labiata*	Czech Republic; Turkey	NA	AJ319636	NA
*Radix lagotis*	Austria, Schönau	NA	AJ319639	NA
*Radix luteola*	Sri Lanka	NA	NA	AF485648
*Radix ovata*	Germany, Tubingen	NA	NA	AF485647
*Radix quadrasi*	Philippines, Luzon	NA	EU556315	U82075
*Radix rubiginosa*	West Java; Malaysia	NA	EU556316	U82076
*Radix *sp. EEAR-Canada-2002	Canada, Manitoba	NA	NA	AF485650
*Radix *sp. EEAR-Philippines-2002	Philippines, Taal Lake	NA	NA	AF485649
*Radix *sp. EEAR-Romania-2002	Romania, Razelm Lake	NA	NA	AF485651
*Stagnicola bonnevillensis*	USA, Utah	NA	NA	AF485655
*Stagnicola caperata*	Canada, Manitoba	AF013140	AF013140	U82077
*Stagnicola catascopium*	USA, Au Sable River (Michigan)	AF013143	AF013143	U82078
*Stagnicola elodes*	USA, Michigan; Canada, Ontario	AF013138	AF013138	AF485652
*Stagnicola elrodi*	USA, Montana	NA	NA	AF485656
*Stagnicola emarginata*	USA, Higgins Lake (Michigan)	AF013142	AF013142	U82081
*Stagnicola *sp. EEAR-Manitoba-2002	Canada, Manitoba	NA	NA	AF485653
*Stagnicola *sp. EEAR-Montana-2002	USA, Montana	NA	NA	AF485654
*Stagnicola *sp. EEAR-Ukraine-2002	Ukraine, Sasyk Lake	NA	NA	AF485662

Species in bold characters were sampled by us. NA: not available.

Genus names are still fluctuating in Lymnaeidae. However, we conserved the names given in GenBank for the sequences retrieved there in order to facilitate comparisons between this and previous studies. For the species we sampled, we adopted the widely accepted genus *Lymnaea *for all species, except for *Galba truncatula*, *Omphiscola glabra*, *Pseudosuccinea columella *and *Radix peregra *(see Table 1).

### DNA Extraction and Polymerase Chain Reaction (PCR) Amplification

Total DNA was isolated from the distal part of the foot. We carefully controlled for trematode presence during dissection in order to avoid exogen DNA. Extractions were performed using the DNeasy Blood and Tissue Kit (Qiagen) according to manufacturers' instructions. The two nuclear internal transcribed spacers (ITS-1 and ITS-2) and the 16 S gene were amplified using published primers [6,42] (Table 2). PCR amplification was performed for each pair of primers in a total volume of 25 μl containing 5 μl of PCR reaction buffer 5×, 2.5 mM MgCl_2_, 200 μM of each dNTP, 10 pmol of each primer, 1 U GoTaq DNA polymerase (Promega), and 2 μl of DNA template. Temperature cycling for the ITS-1 and ITS-2 was as follows: 94°C for 2 min, 94°C for 30 sec, 50°C for 30 sec, 72°C for 30 sec, repeated for 30 cycles, and final extension at 72°C for 7 min. Temperature cycling for the 16 S gene was 95°C for 3 min, 50°C for 2 min, 72°C for 1.5 min, four times at 93°C for 15 sec, 50°C for 8 sec, 72°C for 1.5 min and 25 times at 93°C for 5 sec, 8 sec at 50°C, 72°C for 1 min and final extension at 72°C for 10 min. The amplified products (5 μl) were checked on 1% agarose gels in TAE buffer. DNA sequencing was performed by CoGenics Genome Express (Meylan, France) using PCR-amplified products as templates.

**Table 2:** Sets of primers used in PCR amplifications.

Locus	Forward Primer	Sequence 5' > 3'	Reverse Primer	Sequence 5' > 3'
ITS-1	Lym1657	CTGCCCTTTGTACACACCG	ITS1-Rixo	TGGCTGCGTTCTTCATCG
ITS-2	News2	TGTGTCGATGAAGAACGCAG	ITS2-Rixo	TTCTATGCTTAAATTCAGGGG
16S	16F	CGCCTGTTTATCAAAAACAT	16R	CCGGTCTGAACTCAGATCACGT

### Sequence Alignment and Phylogenetic Analyses

We aligned ITS-1, ITS-2 and 16 S sequences of 50 lymnaeid species and the three outgroups using Prank v. 100311 [43]. Prank is based on an algorithm that can distinguish insertions from deletions and avoid repeated penalization of insertions. Compared to Clustal-W and Muscle, it considerably improves the alignment quality especially in highly variable sequences such as ITSs. Alignments of individual genes were concatenated in a supermatrix with the seqCat.pl v1.0 script [44].

Two different approaches of tree reconstruction, Maximum Likelihood (ML) and Bayesian Inference (BI), were implemented. Analyses were performed by partitioning the supermatrix on the basis of individual loci. ML analyses were conducted using the best-fitting model of sequence evolution. Model selection was based on Akaike's Information Criterion (AIC) using ModelTest 3.7 [45]. ML trees and the corresponding bootstrap supporting values (BP) of the nodes were obtained with PAUP* 4.0b10 [46] using heuristic search with neighbor-joining starting tree, tree bissection-reconnection swapping and 100 bootstrap replicates. BI analyses were performed with MrBayes 3.2 [47]. The tree space was explored using Markov Chain Monte Carlo (MCMC) analyses with random starting trees, five simultaneous, sequentially heated independent chains sampled every 500 trees during five million generations. Suboptimal trees were discarded once the "burn-in" phase was identified and a majority-rule consensus tree, with posterior probability support of nodes (PP), was constructed with the remaining trees.

### Susceptibility to Infection by *Fasciola hepatica *and *F. gigantica*, and the Evolution of the Chromosome Number in Lymnaeidae

We searched available information on susceptibility to infection by *F. hepatica *and *F*. *gigantica*, derived from either analyses of natural populations or from experimental infections, in all species considered using the ISI Web of Science (Thompson-Reuters). We recorded the susceptible or not susceptible status for each species (Table 3). In addition, the haploid number of chromosomes (n) was obtained from previous publications [7,11,31,48].

**Table 3:** Reports of natural or experimental infection of lymnaeids with Fasciola hepatica or F. gigantica.

Intermediate host	Infection by *F. hepatica*	Infection by *F. gigantica*	Refractory to infection
*Austropeplea tomentosa*	[68]		
*Austropeplea *(*Lymnaea*) *viridis*	[69]	[9]	
*Austropeplea ollula*	[9]	[9]	
*Fossaria bulimoides*	[70]		
*Galba truncatula*	[71]	[9]	
*Lymnaea cousini*	[72]		
*Lymnaea *(*Bakerilymnaea*) *cubensis*	[73]		
*Lymnaea diaphana*	[9]		
*Lymnaea fuscus*	[74]		
*Lymnaea humilis*	[75]		
*Lymnaea *(*Radix*) *natalensis*	[76]	[9]	
*Lymnaea neotropica*	[77]		
*Lymnaea *(*Catascopia*) *occulta*	[9]		
*Lymnaea *(*Stagnicola*) *palustris*	[71]		
*Lymnaea *sp. from Colombia	[53]		
*Lymnaea stagnalis*	[78]		
*Stagnicola turricola*	[9]		
(= *Lymnaea palustris turricola*)			
*Lymnaea viatrix*	[79]		
*Omphiscola glabra*	[71]		
*Pseudosuccinea columella*	[73]	[9]	[80]
*Radix peregra*	[71]	[9]	
*Radix auricularia*	[76]	[81]	
*Radix ovata*	[82]		
*Radix labiata*	[83]		
*Radix lagotis*	[76]		
*Radix luteola*		[9]	
*Radix rubiginosa*	[76]	[9]	
*Stagnicola caperata*	[84]		
*Stagnicola elodes*			[73]

## Results

### A Comprehensive Phylogeny of Lymnaeidae

**Figure 1:**
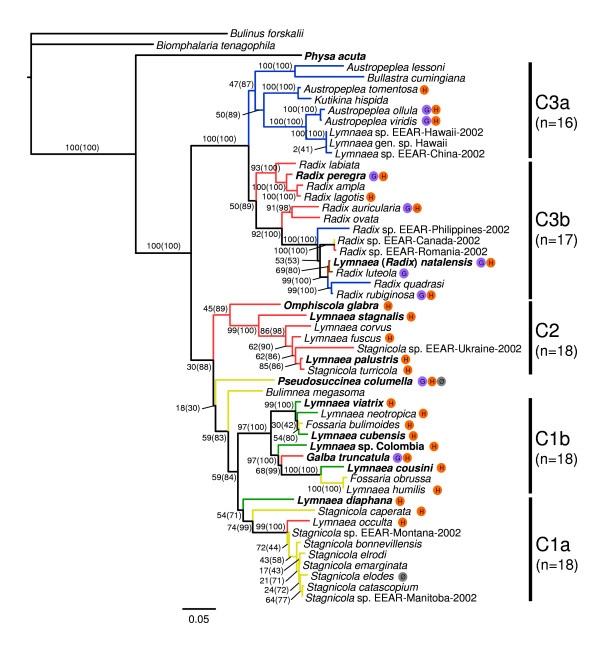
**Phylogeny of the Lymnaeidae**. The tree was obtained by concatenating the 16 S, ITS-1 and ITS-2 sequences, and includes 50 species and three outgroups. Colored branches represent geographic origin; blue = Australasian; red = Eurasia; brown = Africa and Indic ocean; ochre = North America; green = Central and South America. Species naturally or experimentally serving as intermediate hosts of *Fasciola hepatica *(H), *F. gigantica *(G) or refractory to infection (Ø) are shown. (n) is the haploid number of chromosomes. Values on nodes represent bootstrap percentages (BP) and posterior probabilities (PP; given within parentheses). Species sequenced by us are in bold characters.

The ML and BI trees inferred with the alignment of the supermatrix including 50 Lymnaeidae species and three outgroups (5054 aligned sites) were extremely similar, although some node supports varied between the two approaches. The best model describing the evolution of the supermatrix was TVM+I+G (proportion of invariable sites = 0.2298; shape parameter = 0.8159). Overall, the clades obtained here are consistent with previous results [7-9,18,39,41]. Three deeply-rooted clades (hereafter C1, C2 and C3) were detected, basically matching the geographic origin of species (Figure 1). The C1 clade (n = 18) included all American species and two species thought to originate from Europe (*Galba truncatula *and *Lymnaea occulta *= *Catascopia occulta*), although the inclusion of *Pseudosuccinea columella *was only weakly supported (0.18 BP and 0.30 PP). Two highly supported subclades can be recognized within this clade. The first one (C1a) included the South American *L. diaphana*, the North American *Stagnicola caperata*, the European *L. occulta *(= *C. occulta*), and all other North American *Stagnicola*. The second subclade (C1b) grouped the South American *L*. *cousini *with the North American *Fossaria obrussa *and *L. humilis*, on the one hand, and the European *G. truncatula*, Neotropical *L. cubensis *(= *Bakerilymnaea cubensis*), *L. neotropica *and *L. viatrix*, the North American *F. bulimoides *and *Lymnaea *sp. from Colombia, on the other hand.

The C2 clade (n = 18) consisted of exclusively Eurasian species. In this clade, *Omphiscola glabra *(a small-shelled, morphologically distinct species) first diverged from all other species, including most large-bodied species found in Europe (excluding *Radix *spp.). This was followed by divergence of *L. stagnalis*, *L. corvus *(= *S. corvus*), *L. fuscus*, *Stagnicola *sp. EEAR-Ukraine-2002, *L. palustris *(= *S. palustris*) and *S. turricola *(= *L. palustris turricola*). These relationships are identical to those reported in [7,8,18].

The C3 clade contained all Australasian and *Radix *species, including the African *Lymnaea *(*Radix*) *natalensis*. ML and BI analyses indicated that there were two subclades within C3. The first one (C3a) was formed by *Austropeplea lessoni *and *Bullastra cumingiana *(n = 16), *A. tomentosa *and *Kutikina hispidina *(n = 16), and a subclade grouping *A. ollula *and *A*. *viridis *(= *L. viridis*) (n = 16), on the one hand; and *Lymnaea *sp. EEAR-China-2002 and *Lymnaea *sp. EEAR-Hawaii-2002 (n = 18 according to [48] and [11]; n = 16 according to [7]), on the other hand. The second subclade (C3b) consisted of all *Radix *species (n = 17), including *L*. (*R*.) *natalensis*, and included two monophyletic groups. The first one was formed by *Radix labiata *sister to *R. peregra*, *R. ampla *and *R. lagotis*. Note that our samples of *R. peregra *from Southern France clustered within the MOTU2 clade described in [26] with no ambiguity, based on analyses of COI and ITS-1 sequences (results not shown). The second, more recently derived clade, was formed by *R. auricularia *and *R. ovata *which were sister to a clade comprising *Radix *sp. EEAR-Philippines-2002, *Radix *sp. EEAR-Canada-2002 and *Radix *sp. EEAR-Romania-2002, *L*. (*R*.) *natalensis *sister to *R. luteola*, and *R. quadrasi *sister to *R. rubiginosa*.

## Discussion

### A Comprehensive Phylogeny of Lymnaeidae

Recent studies have suggested that the Lymnaeidae contains approximately 100 species [1,2], meaning that our phylogeny represents approximately half the existing diversity of the family. The tree presented in Figure 1 indicates that species cluster by geographic origin in three deep clades. One is almost entirely composed of American species, while the two others are from the Old World. The split between the American C1 clade and Old World C2 clade probably dates back to the opening of the Atlantic Ocean 160-130 million years ago (Mya). This date is reasonable given the fossil record suggests the divergence of Physidae-Lymnaeidae took place near the Jurassic period (~200-145 Mya; [49]).

Concerning the American clade (C1) various aspects should be highlighted. Our study is the first to include *L. diaphana *in a phylogenetic analysis and suggests that this species is sister to the North American *Stagnicola *and *L. occulta *(= *C. occulta*) (clade C1a), although with low support (0.54 BP and 0.71 PP). Baker [50] reported *L. diaphana *in South Dakota, consistent with a possible North American origin. However, the species was originally described from southern South America (Strait of Magellan, Chile; [51]) and is currently found in Argentina, Brazil, Chile, Peru and Uruguay. It could be either that the current distribution of *L. diaphana *in South America is not representative of its North American origin or that the ancestor of clade C1a was originally from South America and then migrated northwards, where it gave origin to the *Stagnicola *of clade C1a. A further point is that *L*. *occulta *(= *C. occulta*) from Poland is unambiguously grouped with this clade and agrees with [41]. This is unexpected as this species is thought to originate from Europe, and was formerly grouped, on the basis of anatomical characters, together with the European *L. palustris *(= *S*. *palustris*) and *S. turricola *(= *L. palustris turricola*), which appear, as expected, in a predominantly European clade (C2). We hypothesize that *L. occulta *was actually introduced in Europe from North American populations (see below). Finally, our results do not contradict the hypothesis that *S. emarginata*, *S. elodes *and *S. catascopium *are conspecifics, as suggested by [6] and [18]. If this is true, *Stagnicola *sp. EEAR-Manitoba-2002 should also be synonymized with these three taxa.

Species with similar shell morphology (*G. truncatula*-like) in the C1b clade cluster together. The only exception is *L. cousini *which clearly belongs to this clade (with maximal BP and PP values) but is morphologically different from all other species in terms of both shell morphology and the anatomy of reproductive organs. Its phylogenetic position suggests the ancestor of clade C1b had a *G. truncatula*-like morphology and the *L. cousini *morphology is a derived character. The inclusion of *G. truncatula *within this clade is unambiguous and agrees with previous results [10]. This species has always been considered a native from the Old World, occurring in Europe, Russia, and North Africa, and to have been recently introduced to South America [32,52]. Our phylogeny suggests that *G. truncatula *represents a branch of an American clade that reached the Old World, where it has evolved and diverged from its American sister species (see below). Interestingly, *Lymnaea *sp. from Colombia, previously described as *G. truncatula *[53], was unambiguously identified as a distinct taxonomic entity, yet to be sequenced. We currently lack sequences and population-genetic studies of several taxa described in North America presenting morphological similarities with *Lymnaea *sp. Therefore, we are not in position to ascertain that this is a new species and to determine whether it is endemic or has been introduced to South America. Thus, we refrain from describing this entity as a new species to avoid adding more noise to the already confusing systematics of the Lymnaeidae.

The second deep clade (C2) consists exclusively of Eurasian species, and species branching agrees [7] and [18]. It does not contradict the hypothesis that *L. palustris *(= *S*. *palustris*) and *S. turricola *(= *L. palustris turricola*) are synonymous, as proposed by [18]. These two taxa indeed only differ by two anatomical characters (preputium length to penis length ratio, and the relative length of the distal part of the prostate) [54].

The third deep clade (C3) includes all species inhabiting Australia and the Indo-Pacific region, as well as *Radix *species. According to this tree, *Austropeplea *is a polyphyletic genus, in agreement with results obtained by [6], [7] and [11]. Remigio [7] suggested monophyly of *Autropeplea *spp., *Bullastra cumingiana*, *Lymnaea *sp. EEAR-China-2002 and *Lymnaea *sp. EEAR-Hawaii-2002. Our results indicate that *Kutikina hispida *and *Lymnaea *gen. sp. from Hawaii are also members of this clade. On the other hand, *Radix *seems to be a monophyletic genus, in agreement with [8], [11], [18] and [26], but in contradiction with results of [6] and [7]. Importantly, *L*. (*R*.) *natalensis*, the only known endemic African species, branched unambiguously within the *Radix *clade, in agreement with [39], sister to *R. luteola *from Sri Lanka. This result confirms that the name *Radix natalensis *better reflects its phyletic relationships than does *Lymnaea natalensis*. Our results do not support the hypothesis that *R. peregra *and *R. ovata *are synonymous [18]. Their synonymy is indeed still a controversial matter [26,38,55]. Alternatively, *R. peregra*, *R. ampla *and *R. lagotis *are closely related taxa, in agreement with [18], and might potentially be conspecifics, although confirmation using mating experiments seems necessary.

In summary (and ignoring recent introductions) we have three old centers of diversification in the Lymnaeidae family: America, Eurasia and the Indo-Pacific region. In the latter, the *Radix *clade diversified and then expanded towards Eurasia and Africa. This is also true of *G. truncatula*, a branch of the American clade that invaded the Old World. In general, the Lymnaeidae morphology has evolved slowly and most species within clades are similar: small-shelled turriform, *G. truncatula*-like in the American clade; large and high-spired shells in the Eurasian *Lymnaea*; and large, rounded or ovate shells in the Indo-Pacific clade, especially in *Radix*. A few branches, including *O. glabra*, and *L. cousini*, have evolved distinctive morphologies that differ from all other lymnaeids. Note, however, that limpet-shaped species (*e.g*., *Lanx*) were not analyzed here, and it is not possible to discuss their phylogenetic position and morphological evolution. If they constitute a separate clade, say C4, our hypothesis of slow morphological evolution would be valid.

Concerning chromosome numbers, [31] and [56] hypothesized that evolution in Lymnaeidae proceeded from low (n = 16) to high (n = 17 and 18) values. Accordingly, *Austropeplea *with 16 chromosome pairs was thought to be the most "primitive" form, followed by *Radix *with 17 pairs and *Stagnicola *with 18 pairs. Our molecular phylogeny contradicts this idea. The ancestral state in Lymnaeidae seems to be n = 18, as it is in other Basommatophoran gastropods (Chilinidae, Lancinae, Latiidae, Planorbidae and Physidae) [57]. Eighteen pairs of chromosomes is likely to be a pleisomorphic character, in agreement with [6] and [7], and thus species of clades C3a (n = 16) and C3b (n = 17) would represent derived rather than ancestral states. Remigio [7] suggested that n = 16 evolved from n = 17. Our results do not contradict this idea, although it is difficult to infer the number of chromosomes for the ancestor of clade C3. In addition, if *Lymnaea *spp. from Hawaii and China have indeed 18 chromosome pairs, as suggested by [11], either a reversion from n = 16 to n = 18 or three independent evolutions to n = 16 would be required (Figure 1).

### Nomenclature in Lymnaeidae

The nomenclature of genera has been one of the most confusing issues in the Lymnaeidae systematics. Most genus names are not fixed and are based more on phenotypic resemblances than on sound evolutionary and phylogenetic considerations. For instance, a single genus was recognized by [58], two by [12], and up to 34 genera by others [13,59-62]. Our results indicate that genera in Lymnaeidae do not reflect phylogenetic relationships, to the notable exception of *Radix *(including *L*. (*R*.) *natalensis*).

The type species of *Lymnaea *is *L. stagnalis *Linnaeus, 1758; the type species of *Stagnicola *Jeffreys, 1830 is *S. palustris *(= *L. palustris*); and the type species of *Omphiscola *Rafinesque, 1819 is *O. glabra*. However, it is clear from our results that these three species belong to the same clade (C2) and that *Lymnaea *is not a monophyletic group. We propose that species of clade C2 should all be called *Lymnaea*, according to the principle of priority of the International Code of Zoological Nomenclature (ICZN). By extension, *Stagnicola *should not be used to name species in clade C1a since the type species belongs to clade C2. Meier-Brook and Bargues [63] suggested including *S. emarginata*, *S. elodes*, *S. catascopium *and *L*. *occulta *within a new genus *Catascopium*, while *S. caperata *would belong to the genus *Hinkleyia *Baker, 1928 [8]. Our phylogeny does not conflict with this nomenclature, although it would seem preferable to identify all species of clade C1a with the same name to reflect the close evolutionary relationships among these species. *Hinkleyia *would be the preferable name according to the ICZN. On the other hand, at least four genera names have been used for species of clade C1b: *Lymnaea *Lamarck, 1799; *Galba *Schrank, 1803; *Fossaria *Westerlund, 1885; and *Bakerilymnaea*. In the light of the present results, it would be preferable to unify nomenclature. According to the ICZN, *Lymnaea *should be the unified name, but given that the type species belongs to clade C2, *Galba *could be a more appropriate name. Finally, as said above, *Austropeplea *Cotton, 1942 is not a monophyletic group, and employing the genus *Kutikina *Ponder and Waterhouse, 1997 (one species: *K. hispida*) seems unjustified on the basis of the current phylogeny. This would also be consistent with results of [11]. It would be preferable to use *Bullastra *Pfeiffer, 1839 for all species of clade C3a to fit the ICZN.

### Unravelling Biological Invasions of Lymnaeidae using the Phylogeny

Although the Lymnaeidae phylogeny presented in Figure 1 matches reasonably well with the geographical origin of samples, some species clustered in clades with different geographic origins stand out and potentially correspond to biological invasions of a more or less recent origin. From an epidemiological standpoint, this is a key issue because some lymnaeid species are more susceptible than others to trematode infection, hence biological invasions can help explain the broad geographic distribution of fascioliasis and in determining the risks for veterinary and public health (see e.g. [64]).

First, one of our most puzzling results concerns the origin of *G. truncatula *Müller, 1774, the main vector of fascioliasis in the Old World. The idea that this species is native to Europe, as it was described from Germany, is widely accepted [65]. However, a very different picture emerges from our phylogeny: it is the only European species branching with the wholly American clade C1b. This strongly suggests that America is where *G. truncatula *originated. This is consistent with its detection in Alaska and the Yukon territory [50]. It is possible the current distribution of *G. truncatula *in North America is broader, but has remained cryptic because it has been confounded with other taxa. Indeed, Hubendick noted that, "it is a matter of some doubt whether *Lymnaea humilis *in North America is a distinct species or is specifically connected to *L. truncatula*" (= *G. truncatula*) [66]. It could be that some populations of *L. humilis *in reality correspond to *G. truncatula*. At least 10 species of Lymnaeidae from North America placed in synonymy of *L. humilis *by [12], but considered as valid by [13], present conchological similarities with *G. truncatula*: *L. galbana *Say, 1825; *L*. *modicella *Say, 1825; *L. obrussa *Say, 1825; *L. parva *Lea, 1841; *L. exigua *Lea, 1841; *L. rustica *Lea, 1841; *L. tazevelliana *Wolf, 1869; *L. dalli *Baker, 1906; *L. cyclostoma *Walker, 1908; and *L. peninsulae *Walker, 1908. Unfortunately, neither detailed anatomical descriptions nor molecular data are available in any of these taxa. Sampling and sequencing of morphologically similar North American taxa could shed light on this question.

Second, as mentioned above, *L. occulta *(= *C. occulta*) is the only species from Europe clustering within clade C2 and seems to correspond to a passage from North America to Europe. As stated by [8] (and references therein), *L. occulta *is distributed in eastern Germany, Poland, the former Czechoslovakia, the former Yugoslavia, Ukraine, Sweden, and some rivers in the delta of Lake Baikal. It is hypothesized that the species could reach the far east Asia where it could have been confounded with other stagnicoline species because of similarity in shell morphology [12]. In any case, it seems clear that *L. occulta *has its origins in America.

Finally, the two *Radix *sp. are likely introductions originating from the Indo-Pacific area to Canada and Romania. The fact that these two sister taxa (potentially the same species) are found in distant geographic locations is indicative of recent introductions.

### The Relationships between Lymnaeidae and *Fasciola hepatica *and *F. gigantica*

From an epidemiological standpoint, digenetic trematodes show marked specificity for their intermediate hosts but can infect a broad spectrum of definite hosts. Usually, species are oioxenous (one parasite species: one snail species) or stenoxenous (one parasite species: a few, closely related snail species) [67]. This is because when the infectious form of the parasite (the miracidium) enters a snail, it must encounter an internal physiological and biochemical environment that supports its complete development. However, the case of *F*. *hepatica *seems to be different. Our results show that lymnaeid species serving as intermediate hosts of this trematode are widely distributed across the phylogeny (Figure 1). Basically, all clades contain species that have proven to be naturally or experimentally infected with this parasite. Only a few species have been shown to be resistant to infection (Table 3). In contrast, species involved in *F. gigantica *transmission are more clustered in the phylogeny (Figure 1). Although not all species are equally susceptible to infection by *F. hepatica*, the broad capacity of this parasite to infect phylogenetically distant species is remarkable. The Lymnaeidae-*Fasciola *system differs from the well known Planorbidae-*Schistosoma *system, in which each trematode species infects a narrow group of hosts that share a close phylogenetic relationship [4]. The behavior of *F. hepatica *with respect to Lymnaeidae is of paramount importance in epidemiology control programs: rather than focusing on a single, or a handful of snail species, fascioliasis control programs should cover a broader spectrum of intermediate hosts that inhabit diverse habitats and ecological conditions. Our results confirm that the presence of *F. hepatica *in all continents is strongly favored by its capacity to infect local lymnaeids. Based on these results, it seems that all geographic regions in the world are exposed to the epidemiological risk of fascioliasis.

## Conclusion

At least four conclusions can be drawn from this study. First, combining information from different genes (supermatrix) is a robust approach to reconstruct the evolutionary history of the Lymnaeidae. Our results indicate that members of this family diverged in three deeply-rooted clades corresponding to the geographic origin of species (America, Eurasia and the Indo-Pacific region). Our phylogeny allowed us to pinpoint discordances between ancestral and current geographic distributions of some species, potentially indicating more or less recent biological invasions. Transfers from America to Eurasia are suggested for *G*. *truncatula *and *L. occulta *(= *C. occulta*), as well as passages from the Indo-Pacific to Europe and North America for *Radix *sp. However, sampling and sequencing efforts remain to be done especially in the Palaearctic and Nearctic regions in which the family diversity is thought to be the largest. This would help resolve the weakly supported relationships (*e.g*., *P*. *columella*), determine the pace of morphological evolution, establish taxonomic synonymy, and determine the phylogenetic relevance of poorly known genera (*e.g*., *Acella*, *Lantzia*, *Lanx *and *Myxas*). Second, with the exception of *Radix *(including the African *L*. (*R*.) *natalensis*), genus names in Lymnaeidae do not reflect the phyletic relationships among species. The group taxonomy should be reconsidered to gain some biological meaning. Third, the number of chromosomes in Lymnaeidae has evolved from an ancestral state of 18 pairs to a derived 17 and 16 pairs. Finally, while the intermediate hosts of *F. gigantica *are basically restricted to clade C3, *F. hepatica *is able to infect species from all main clades (C1, C2 and C3). This suggests that the cosmopolitan distribution of *F. hepatica *is largely favored by its capacity to infect local lymnaeids, and highlights the importance of the correct identification of intermediate-host species in fascioliasis control programs.

## Authors' contributions

ACC and SHB designed the study. ACC, JSE, PDavid, PJ and JPP sampled snails. ACC and PDurand carried out experiments. ACC and JSE performed analyses. All authors discussed the results and wrote the paper.
